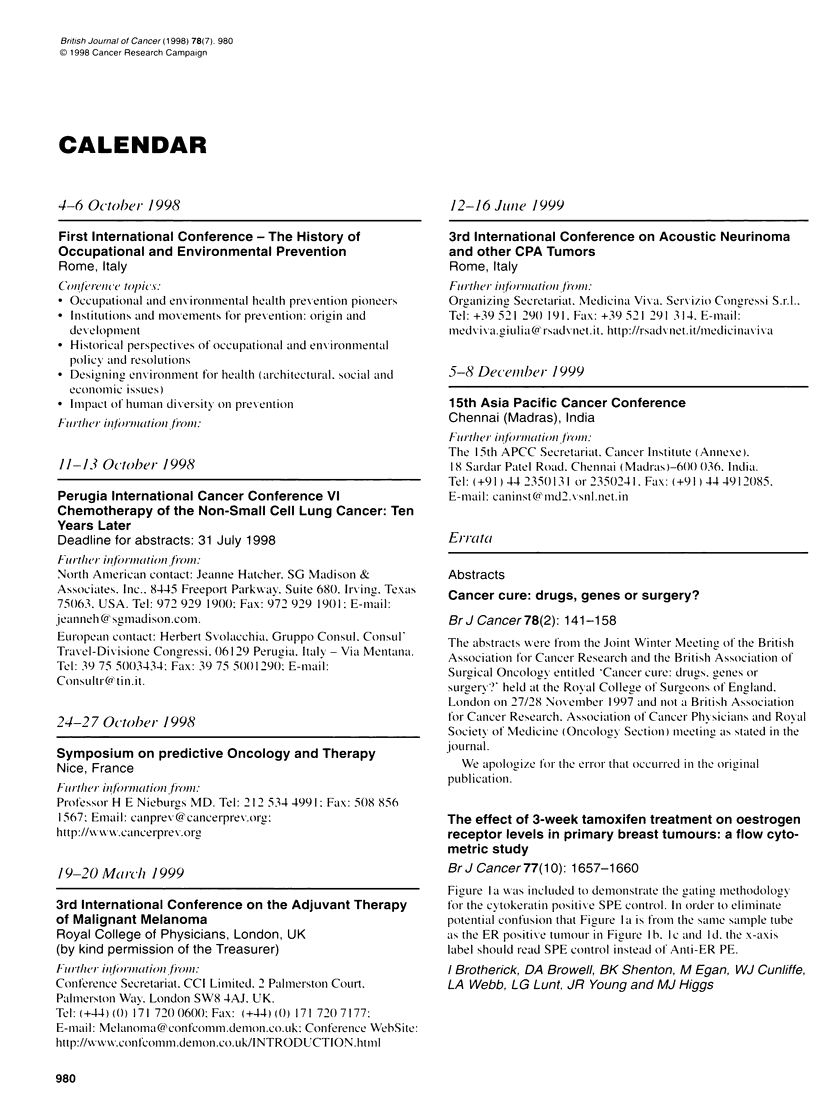# The effect of 3-week tamoxifen treatment on oestrogen receptor levels in primary breast tumours: a flow cytometric study

**Published:** 1998-10

**Authors:** 


**The effect of 3-week tamoxifen treatment on oestrogen receptor levels in primary breast tumours: a flow cytometric study**

*Br J Cancer* **77**(10): 1657–1660

Figure 1a was included to demonstrate the gating methodology for the cytokeratin positive SPE control. In order to eliminate potential confusion that Figure 1a is from the same sample tube as the ER positive tumour in Figure 1b, 1c and 1d, the x-axis label should read SPE control instead of Anti-ER PE.

*I Brotherick, DA Browell, BK Shenton, M Egan, WJ Cunliffe, LA Webb, LG Lunt, JR Young and MJ Higgs*